# Transition from independent midwifery care to Early Prevention Network Services in Germany – a cross-sectional survey

**DOI:** 10.1186/s12913-026-14414-4

**Published:** 2026-03-24

**Authors:** Anne C. Hallet, Claudia Oblasser, Loukia M. Spineli, Mechthild M. Gross

**Affiliations:** 1 Midwifery Research and Education Unit, Hannover Medical School, Hannover, Germany; 2 IMC University of Applied Sciences Krems, Krems, Austria

**Keywords:** Midwifery, Collaboration, Family health services, Transition of care, Continuity of care

## Abstract

**Objective:**

Midwives in Germany offer various care options, including independent midwifery care funded by statutory health insurance and care in Early Prevention Networks with a family-centered focus. Consequently, independent midwives may transfer families who need additional care to Early Prevention Network Services, with possible implications for continuity of care. We aimed to determine the frequency of transitions of care, related communication, midwives’ knowledge and satisfaction, and reasons for not participating in transitions of care.

**Methods:**

A cross-sectional survey among independent midwives in Germany was conducted. Potential participants were invited via midwifery organisations to complete an online questionnaire, with 292 participating. Data analysis was carried out using IBM SPSS 27 and Microsoft Excel. Thirteen characteristics of the care and carers were summarised using descriptive statistics and graphs.

**Results:**

Less than half (*n* = 141; 49%) of the responding midwives reported at least one transition of care during the past 12 months. In this group, 36% (*n* = 86) answers stated that communication with early prevention occurred indirectly via the families. In the group without a transition of care (*n* = 150; 51%), the main reason for this absence was a lack of need by the families (*n* = 120; 63%). However, most midwives were satisfied or very satisfied with the collaboration (*n* = 109; 44%).

**Conclusion:**

The findings indicate a need for further research on strategies such as co-location to improve this collaboration, although they cannot be generalised. Continuity of care deserves attention when collaboratively caring for vulnerable families.

**Supplementary Information:**

The online version contains supplementary material available at 10.1186/s12913-026-14414-4.

## Background

Midwives play an essential role in perinatal healthcare. As pointed out in the ‘Framework for Quality Maternal and Newborn Care’ [1], the scope of midwifery encompasses health education for pregnant and postpartum women and childbearing people, as well as the promotion of physiological processes and the detection and handling of complications. Midwives work in various settings, including hospitals, communities and homes [2]. In many countries, midwives not only provide essential perinatal healthcare services but are also integrated into more comprehensive preventive family healthcare options that extend into early childhood [3, 4]. These services are available in various forms. In some countries, such as the United Kingdom, universal services are provided to the general population through health visitors [3, 5]. However, in other countries such as Germany, the services are more targeted at vulnerable populations. This means that perinatal care providers actively suggest and initiate additional care through preventive services when indicated, as described by Eickhorst and colleagues [6].

### Early Prevention Network Services

The preventive services in Germany are known as Early Prevention Network Services and were established in 2006 as collaborations between perinatal professionals from the health and social sectors [7]. They are primarily managed and coordinated regionally by Youth Welfare Offices, and in some cases by health authorities or social institutions [8]. Early Prevention Services are regulated by the Federal Child Protection Act and funded by a federal foundation [7]. The actual perinatal care is delivered by family midwives and family nurses, thus midwives and nurses with an additional psychosocial qualification [9, 10]. Family midwives can visit families from the beginning of pregnancy until the child’s first birthday [9], whereas family nurses’ care starts from birth and can continue until the child is 3 years of age [10]. The main target group is the perinatal population with psychosocial burdens, such as mental health conditions, poverty, single or unplanned parenthood, regulatory problems of the infant, high caregiver stress, and/or dysfunctional families [7]. A focus in the work of family midwives and nurses is the comprehensive care not only for mother and child, but also for the entire family in the course of pregnancy and early childhood [9, 10]. The work of family midwives and nurses is mainly based on individual home visits for check-ups, counselling, and administrative support, but also includes accompaniment to appointments and case management, often in collaboration with other professionals [11].

The uptake of the service has been evaluated in a national survey of paediatric practices by Eickhorst and colleagues [6]: 12.9% (95% CI 12.0–14.0) of families with children up to 3 years of age used the services of family midwives. Moreover, different studies estimated the number of families needing psychosocial support as 7.8–29.1%, depending on the underlying criteria [6, 12, 13].

### Independent midwifery care

**Fig. 1:**
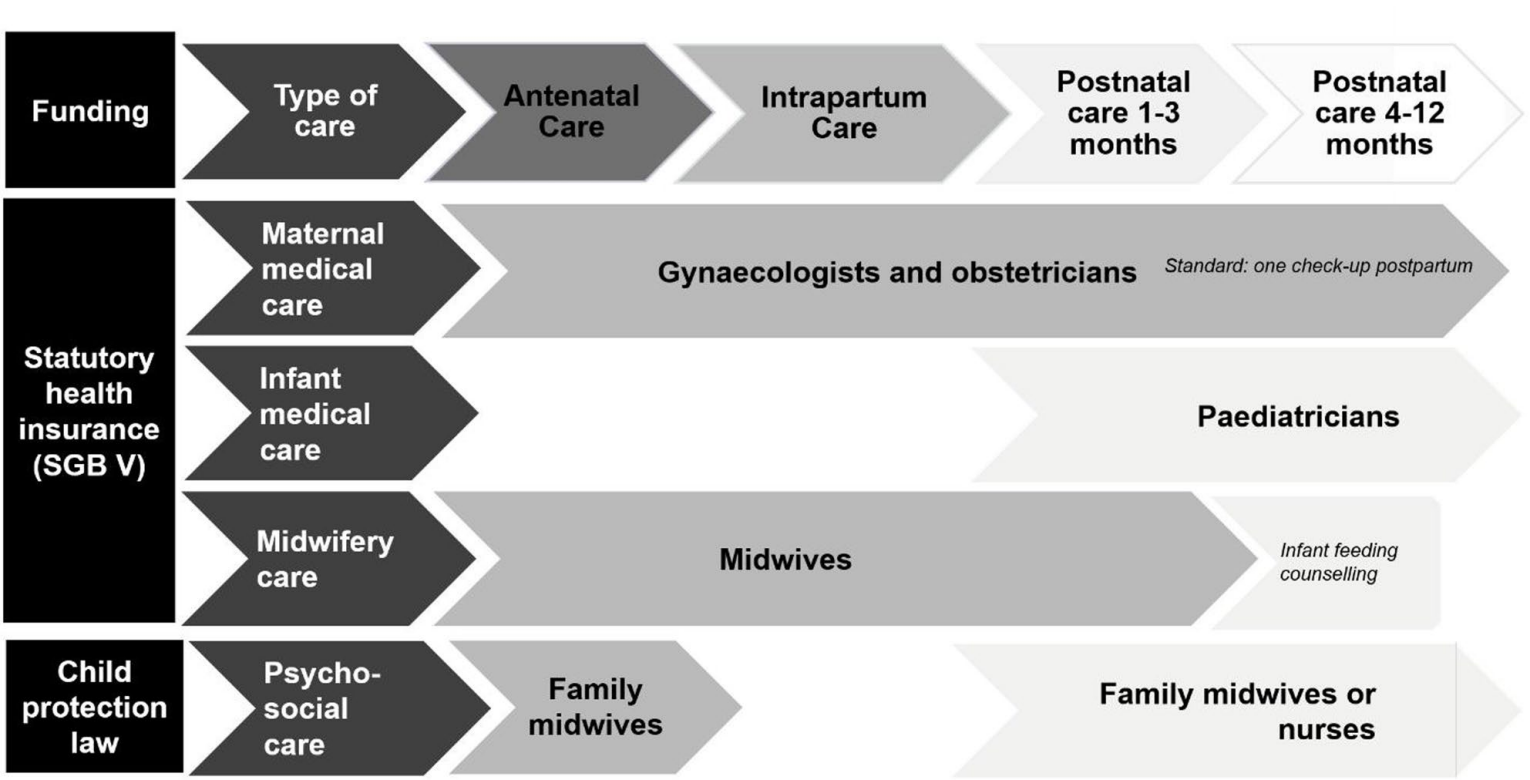
Perinatal healthcare in Germany. *Note*: Adapted from van Minde et al. [4]

At the same time, independent midwifery care is included in the benefits of statutory health insurance so that all insured families can use their services, such as antenatal and postnatal care, if they actively seek a midwife [14]. An overview of healthcare options during the perinatal period in Germany is presented in Fig. 1. Postnatal midwifery care, mostly by independent midwives, is very common, with more than 90% of the families reporting in a national study to have received this form of care [15]. However, the number of appointments is limited: for instance, after childbirth, daily visits in the first 10 days and a maximum of 16 appointments in the first 12 weeks are reimbursed, with possible prolongation for counselling on feeding or on medical advice [14].

### Collaboration between midwifery care and Early Prevention

Studies from several countries [4, 5, 16–18] emphasise improving the collaboration between midwives and professionals from preventive maternal and child health services, including a systematic review by Aquino and colleagues [3]. These authors reported different facilitators for interprofessional collaboration, including interdisciplinary meetings, local coordinators, an atmosphere of trust and respect, and good communication, as opposed to barriers such as the need for more knowledge about the roles and shortages of time and staff. Other studies found that healthcare professionals in the UK, Australia and the Netherlands reported problems such as fragmented services [18, 19] and incomplete handovers [4, 20]. For Germany, a survey from 2017 among local coordinators of Early Prevention Networks [8] found that independent midwives were integrated into 70.0% of these networks. However, the number of midwives in each network was not quantified. In this study, the quality of the collaboration was rated as a mean value of 2.5 on a five-point Likert scale without any statement of the respective reasons.

### Transitions of care

Family midwives and nurses offer care outside the benefits of the statutory health insurance, and independent midwives may hand their clients over to them or provide care simultaneously [11, 21]. This can be seen as a transition of care: a point when a client or patient moves from one location or professional to another within the healthcare system [22].

In a targeted approach, these transitions are of particular importance. Internationally, they have been repeatedly identified as vulnerable points because they can interrupt the continuity of care [16] and its multiple benefits, which have been shown in a Cochrane review [23]. More precisely, continuity of care is at stake in all three aspects that Reid and colleagues [24] have analysed: continuity of information, management and relation. Homer and colleagues [18] found that, for Australia, communication at transitions of care tends to be heterogeneous, ranging from contact via coordinators to handover on demand to impersonal contact, with the latter being the dominant form. However, international comparisons are challenging because of the targeted approach in Germany.

In a study from Eastern Germany, Ayerle and colleagues [21] surveyed midwives and reported a mean of three transitions of care per month. They also found a need for more knowledge on Early Prevention. In addition, informal contacts around transitions of care and lack of integration in Early Prevention Networks have been reported as common in a study including problem-centred interviews with 27 independent midwives by Schlüter-Cruse [25].

Apart from that, little is known about the number of transitions of care from independent midwives to Early Prevention services in Germany and their related characteristics, even though the importance of this collaboration has been emphasised by service users [26].

### Research questions

To address these gaps in knowledge, we focused on the following research questions:

- How often do independent midwives transfer their clients to family midwives and nurses?
- What are the reasons why independent midwives do not participate in these transitions of care, if applicable?
- How does communication occur during transitions of care?
- How do independent midwives self-assess their knowledge of Early Prevention and their satisfaction with the collaboration?

## Materials and methods

We conducted an online cross-sectional survey among independent midwives in Germany. Independent midwives in Germany are self-employed and primarily bill their services through the contractual partnership of their professional organisations with health insurance companies [14]. According to reports, the German Midwives Association, as the largest organisation, had 14,075 active members in independent perinatal care at the beginning of 2021 [27].

Because independent midwives may offer their services simultaneously via statutory health insurance and work as family midwives, we suggested that this might influence their collaboration with Early Prevention Network Services and chose to exclude those who also work as family midwives. All participants had to confirm they did not practice with respect to Early Prevention before starting the survey.

### The questionnaire

A transition of care was defined as a case in which the independent midwives themselves contacted the Early Prevention Network Services to obtain care from family midwives or nurses, or they motivated the family to do so. Contact was made directly with the respective healthcare professionals or indirectly via a coordinator of the Early Prevention Network Services. The independent midwives were also asked how many families they cared for on a self-employed basis in the past 2 and 12 months.

Since no validated instruments existed on this specific topic, we developed a questionnaire on the subject in the German language (see English translation by authors in supplementary material S1), while taking previous studies into account [3, 18, 21, 25]. The questionnaire consisted of 13–18 items, depending on how the participants answered two filter questions: if a transition of care took place in the past 12 months, and if an Early Prevention Network was available in the municipality where the independent midwife was practising. The information about the transitions of care was also assessed within two time frames, in the last 2 and in the last 12 months. This was done to assess whether personal circumstances or difficulties to recall might have played a role.

The four main areas of the instrument were: the number of families cared for and the number of transitions of care; the characteristics of local Early Prevention Networks, knowledge around Early Prevention Services and related data protection procedures; satisfaction with the collaboration; and sociodemographic characteristics. There were six open and nine closed questions. Three of the latter had options to add free text. The levels of knowledge and satisfaction could be self-assessed on three different five-point Likert scales ranging from ‘very good’ to ‘very bad’ and ‘very satisfied’ to ’very dissatisfied’.

### Ethical aspects

This study was conducted in accordance with the Declaration of Helsinki, and the ethics committee of Hannover Medical School approved this study before its initiation (no. 9981_BO_K_2021). During data collection, an expansion of the recruitment strategies became necessary and was also approved. Participation in this study was voluntary. Potential participants were informed about the study, data protection, and their associated rights, including that participation could be discontinued at any time without giving reasons. Furthermore, explicit digital consent had to be given to start the survey.

No personal data that would allow the identification of participants was collected. The encrypted responses were stored at the SoSci Survey provider in Germany and deleted after 12 months. After data collection, the first author downloaded and stored the data on a protected device. It will only be used for this study, kept for 10 years and then deleted.

### Piloting, sampling and data collection

We piloted the survey with 21 midwives to ensure comprehensibility and face validity in October 2021, and applied minor adjustments to the questionnaire, such as adding a definition for ‘Early Prevention Network’ and one additional response option. Afterwards, we asked the federal–state associations of the German Midwives Association to distribute the invitation to the survey in their newsletters in November 2021. When it became apparent that this only led to moderate participation, recruitment was expanded to district midwifery associations, local midwife networks and midwifery groups on social media (see supplementary S2). This recruitment strategy resulted in a convenience sample.

We cannot quantify how many potential participants were reached by the invitation to the survey and thus cannot calculate a response rate. However, we know that the link to the web-based survey was accessed 791 times. One of the 293 completed surveys had to be excluded from analysis because they reported working as a family midwife, so that finally, 292 independent midwives took part.

### Data analysis

The dataset was screened for errors and implausible values. Four midwives inconsistently answered the question on the number of transitions of care and the corresponding filter question. After reviewing these observations, a more detailed numerical response was adopted, with responses that incorrectly answered the filter question considered as ‘missing’.

We summarised a total of 13 variables using descriptive statistics. Categorical variables were reported as absolute and relative frequencies (%). Continuous variables were described using measures of central tendency (i.e. mean and median) and dispersion tendency (i.e. standard deviation, interquartile range, minimum and maximum). We also reported the size of missing data per variable. Statistical analysis was performed using IBM SPSS Statistics 27.

A structuring content analysis according to Kuckartz and Rädiker [28] was conducted to analyse the free-text answers. For this purpose, the free text was coded into pre-existing and inductively created categories in Microsoft Excel, which were then quantified and used for data analysis of the respective variables.

## Results

### Characteristics of independent midwives

Of the 292 independent midwives, most (*n* = 261, 89.4%) were recruited via regional midwifery organisations. The remaining 31 (10.6%) professionals joined the survey after the invitation was sent via midwifery groups on social media and to a national midwifery society. The participants’ ages ranged from 22 to 70 years (median 43.0), and they had a broad range of professional experience, ranging from 1 to 51 years (median 17.0; Table 1). The participants accessed the survey via different regional and supraregional midwifery organisations or the web (see supplementary material S2).

**Table 1:** Participants’ professional and care characteristics (*N* = 292)

Characteristic	*n*	Missing (%)	Mean (SD)	Median (IQR)	Min–Max
Age (years)	276	16 (5.5)	43.7 (10.9)	43.0 (35.0–53.0)	22–70
Professional experience (years)	276	16 (5.5)	18.1 (10.8)	17.0 (10.0–26.8)	1–51
Families cared for in past 12 months	292	0 (0.0)	55.7 (44.5)	46.5 (25.0–70.0)	0–350
*Midwives without care transition*	150	0 (0.0)	51.2 (47.7)	35.5 (20.0–70.0)	0–350
*Midwives with care transition*	141	0 (0.0)	60.4 (40.6)	50.0 (36.0–70.0)	0–250
*Missing*	1	–	–	–	–
Transitions of care in past 12 months	291	1 (0.3)	1.3 (1.9)	0.0 (0.0–2.0)	0–15

IQR: interquartile range; Min: minimum; Max: maximum; SD: standard deviation

About half of the midwives (*n* = 135) reported additional qualifications, such as mentoring (*n* = 60; 31.7% of the 189 responses, with multiple answers possible), an academic degree (Bachelor, Master or higher; *n* = 55; 29.1%), family midwife (*n* = 32; 16.9%) or lead midwife (*n* = 13; 6.39%). The 45 open responses with a mean response length of 2.49 words (minimum, 1; maximum, 13) were coded into categories, some into the options above and some into newly created ones, such as counselling, lactation consultant (both *n* = 9; 4.8%) and others (*n* = 11, 5.8%).

The size of the municipalities where the midwives mainly worked varied: more than a quarter (*n* = 84; 30.0%) were based in medium-sized cities (20,000–99,999 inhabitants), and about a third (*n* = 100; 35.7%) worked in larger cities. Small towns with 5000–19,999 inhabitants were reported as the main work area for one out of five participants (*n* = 51; 18.2%), while 45 participants (16.1%) worked in rural communities, with 12 missing answers. We have added a table in the additional material (S3) to illustrate the frequency of transitions of care by municipality size. In 10.0% of the responses (*n* = 28), the midwives reported that there was no Early Prevention Network in the municipality where they worked most frequently. However, most (*n* = 251; 90.0%) reported one available, with 13 missing answers.

### Number of families cared for and transitions of care

**Fig. 2:**
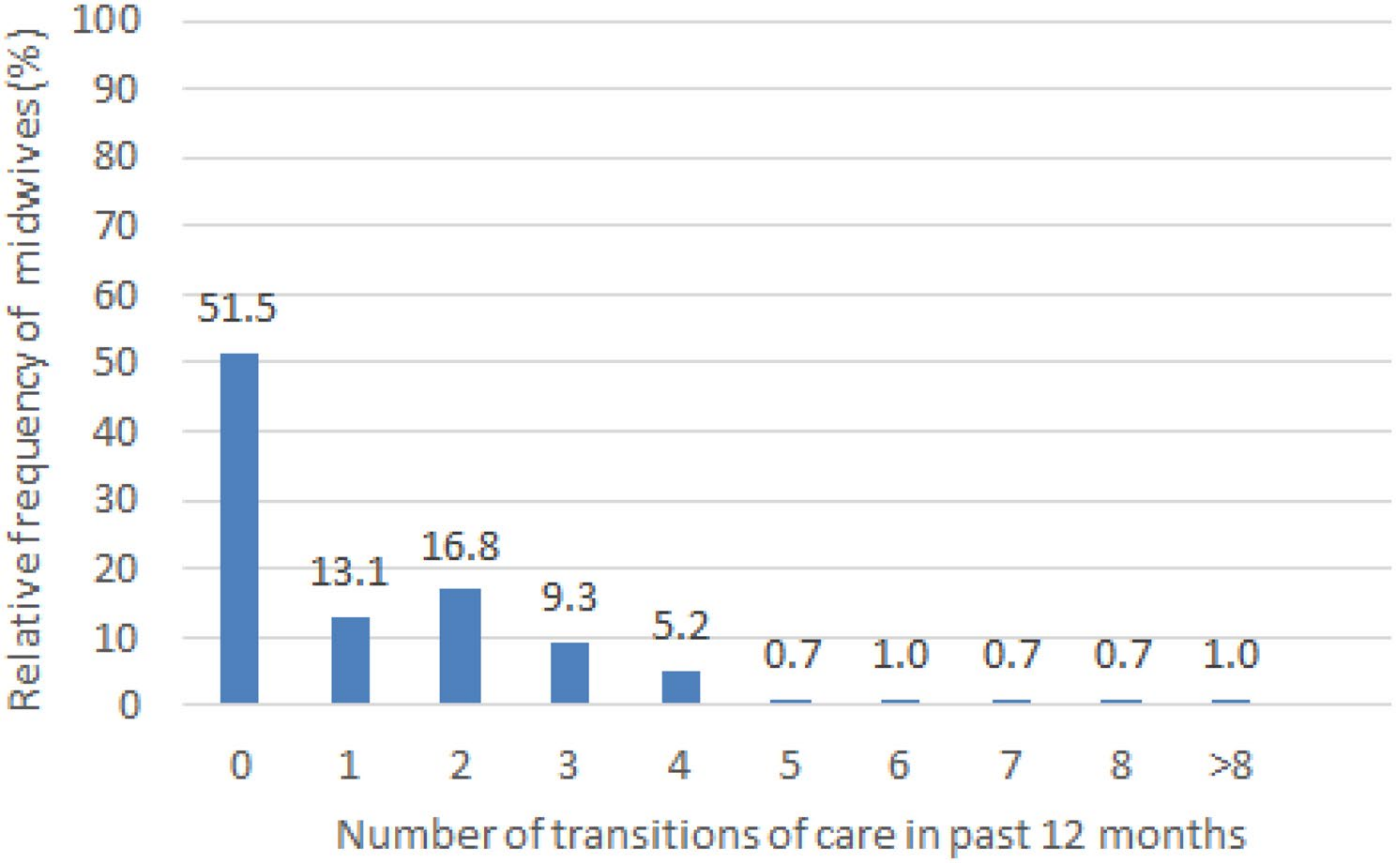
Frequencies of transition of care (*n* = 291)

The midwives cared for a mean of 55.7 families during pregnancy, birth and postpartum over the past 12 months and transferred a mean of 1.3 families during this time (Table 1). Many midwives (*n* = 150, 51.5%; Fig. 2) reported not having participated in any transition of care, with one missing observation. The remaining 141 (48.5%) midwives stated they had performed at least one transition of care (Fig. 2). Overall, midwives without transitions of care looked after fewer families than those with at least one transition of care. Specifically, midwives without performing any transition of care cared for 7675 families (median = 35.5; range = 0–350) in the previous 12 months, while midwives with at least one transition of care cared for 8518 families (median = 50.0; range = 0–250). The absolute number of transitions of care was 372 in 16,273 cases, which corresponds to a relative frequency of 2.3%. We also added data on transitions of care in the past 2 months to the supplementary S4.

### Reasons for the absence of transition of care

**Fig. 3:**
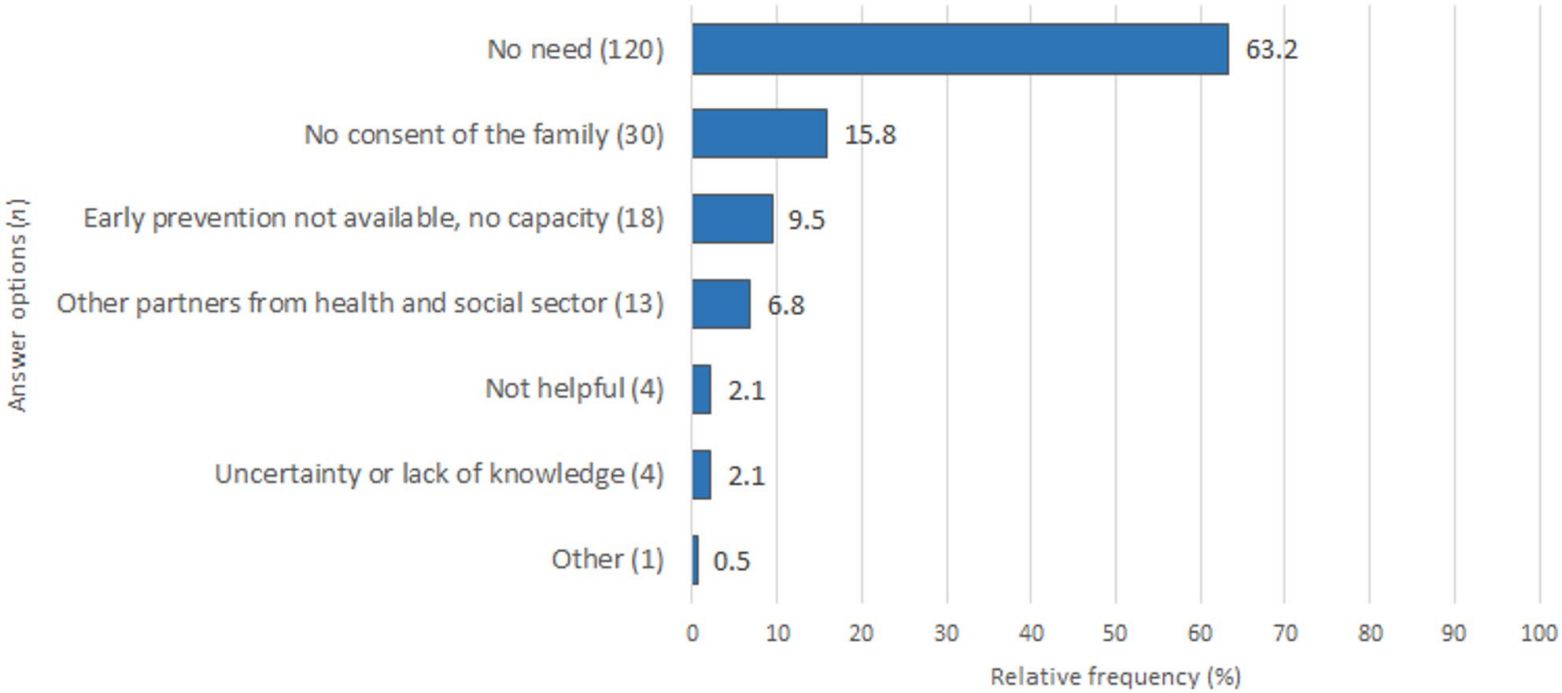
Reasons for the absence of transition of care. Note: Multiple answers are possible, relative frequency based on the total number of responses (n = 190)

Of the 150 midwives without a transition of care, 143 respondents answered the question about the respective reasons. Of the 190 answers, most (*n* = 120; 63.2%) pertained to the lack of need by families as the main reason for the absence of transition of care (Fig. 3). Notably, 15.8% (*n* = 30) of the answers referred to a lack of consent from the family. Approximately 7% (*n* = 13) reported having worked with other partners from the social or healthcare sector, and around 1 in 10 (*n* = 18) expressed difficulties accessing Early Prevention Network Services. Four responses referred to family midwives or nurses not being seen as helpful. Based on the results of the free-text analysis of 12 responses with a mean length of 13.33 words (minimum, 4; maximum, 33), the category of insecurity or lack of information was developed, and four responses (2.1%) were included. The remaining eight free-text responses were coded into pre-defined categories.

### Communication at transitions of care

**Fig. 4:**
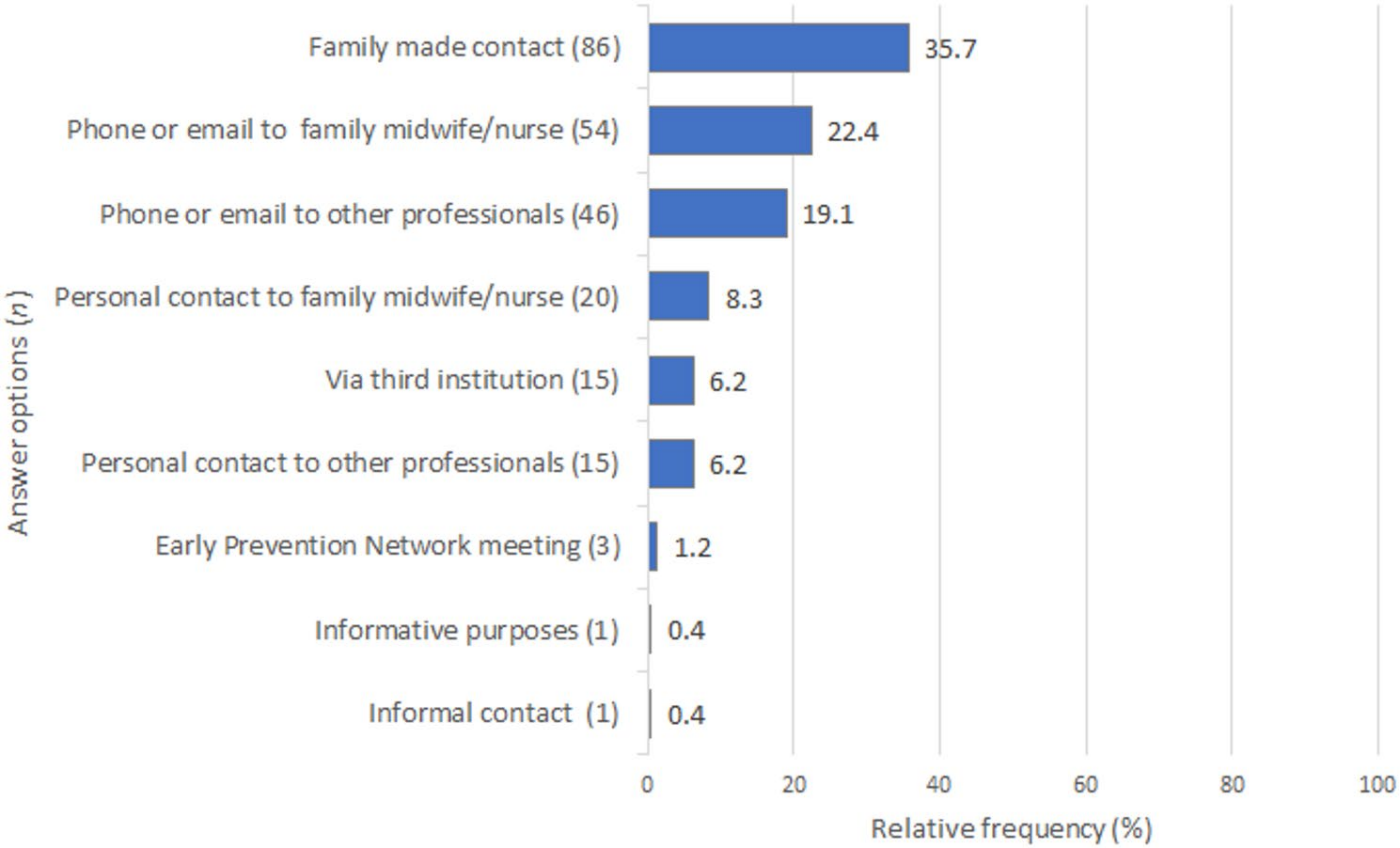
Communication at transition of care. *Note*: Multiple answers possible, relative frequency based on the total number of responses (*n* = 241)

Participants who reported at least one transition of care were asked about related communication (seven missing answers). As shown in Fig. 4, contact was stated to occur most often indirectly. About one-third of the answers stated that families made contact with Early Prevention Network Services themselves. Likewise, the answer options on communication media, such as phone or email to a family midwife/nurse or other professionals, were selected more frequently (22.4%, *n* = 54 and 19.1%, *n* = 46, respectively), than direct and personal contact with family midwife/nurse (8.3%, *n* = 20) and 6.2% (*n* = 15) with other professionals. Some communication occurred via third parties, and only three answers referred to contact via meetings organised by Early Prevention Networks. Additionally, three out of five free-text responses (mean response length 6.6 words: minimum, 3; maximum, 50) were coded into existing categories. One participant reported only contacting Early Prevention to obtain information, rather than for a transition of care, and another referred to informal contact via word of mouth.

### Participation in network meetings, knowledge and satisfaction

Approximately one in five midwives (*n* = 48; 19.1%) with an Early Prevention Network in the municipality where they mostly worked (*n* = 251) reported attending network meetings in the last 12 months.

**Fig. 5:**
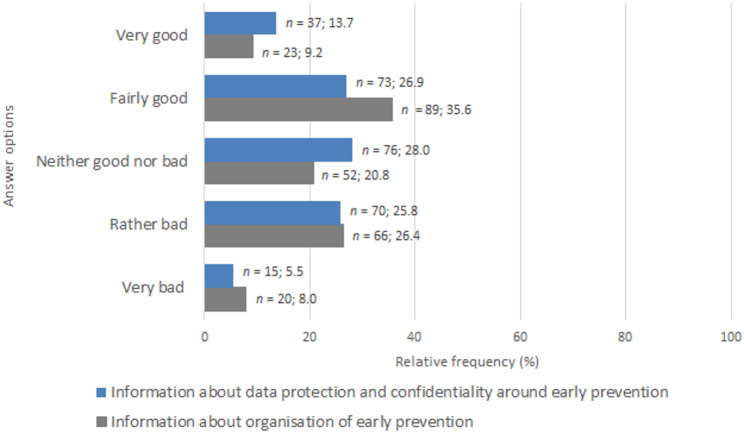
Level of information about data protection/confidentiality* and organisation**. *Note*: Relative frequency based on non-missing observations. *n = 271, 21 missing **n = 252, 1 missing

More detailed data on collaboration were collected for those participants with an Early Prevention Network in their professional area. A mixed picture emerged when assessing how well the midwives felt informed about the organisation of Early Prevention (Fig. 5). Most midwives described their level of information as rather good (*n* = 89; 35.6%), followed by rather bad (*n* = 66; 26.4%) and neither good nor bad (*n* = 52; 20.8%). Only 9.2% (*n* = 23) and 8.0% (*n* = 20) of midwives considered their knowledge to be very good or very bad, respectively. In contrast, the level of information about data protection and confidentiality based on the non-missing observations of the whole sample was relatively balanced for rather good (*n* = 73; 26.9%), neither good nor bad (*n* = 76; 28.0%) and rather bad knowledge (*n* = 70; 25.8%), followed by very good (*n* = 37; 13.7%) and very bad (*n* = 15; 5.5%).

Most midwives with an available network (*n* = 251) knew the statutory body of Early Prevention Networks in Germany. The majority (*n* = 118; 48.4%) named the Youth Welfare Office as the responsible agency, with an independent agency stated in 16.0% of cases (*n* = 39). In comparison, 34 midwives (13.9%) named public health services. About one-fifth (*n* = 53; 21.7%) chose the ‘I do not know’ option; seven answers were missing.

These midwives reported a relatively positive satisfaction with the collaboration. Although the majority were neither satisfied nor dissatisfied (*n* = 98; 39.8%), 32.9% (*n* = 81) described themselves as satisfied and 11.4% (*n* = 28) as very satisfied. Around 13.0% (*n* = 32) were dissatisfied, and 2.8% (*n* = 7) described themselves as very dissatisfied. Five answers were missing.

## Discussion

### Frequencies of transitions of care

The absolute frequency of transitions of care per midwife in Germany (on average 1.3 for the last 12 months) was lower than the mean of three transitions of care per month reported elsewhere [19]. However, we do not know their precise definition of their outcome. In contrast, our results are comparable with those of Schlüter-Cruse [25], who found that 16 of 27 interviewed midwives had contacts with Early Prevention Network Services. Compared with the relative frequency of 2.3% transitions of care that we found, 22% of the families would benefit from care through Early Prevention services, according to independent midwives in one federal state [21]. When looking at the distribution of the number of transitions of care (Fig. 2), it appears to be right-skewed: While a bit less than 50% of our participants reported one or more transitions of care, we also found that the remaining midwives had not participated in at least one transition of care in the previous 12 months.

### Reasons for not initiating transitions of care

Three out of five answers stated a lack of need for further care as a reason. Because we did not assess the psychosocial needs of the families the midwives cared for in our study, we cannot conclude whether there is a need for improved psychosocial risk assessment as has been suggested for paediatricians [8].

The second most common option, the families’ lack of consent, again highlights the important role of an assessment of the families’ needs. We would like to emphasise that the refusal of consent is a fundamental right of the family and must be respected. We also did not collect any data on the family’s risk factors, so we cannot draw any conclusions about the individual situations. As a general consideration, extreme cases with very high psychosocial risk factors could require an assessment of the risk of child endangerment. This requires appropriate training and exchange with other professionals. It is possible that not all midwives are aware of their right to seek advice from the Youth Welfare Office without disclosing the family’s data, as stated in the Child Protection Act.

### Communication around transitions of care

However, even among those midwives with transitions of care, different forms of communication could be observed. The respective communication was often impersonal. About a third of the responses stated that it was mainly the family who established contact with Early Prevention Network Services. These impersonal contacts have been described elsewhere and are seen as inhibiting collaboration [4, 18]. Furthermore, many midwives reported communicating with other professionals or institutions, such as coordinators. However, even if coordinating professionals can contribute to better care [3, 4, 29], the question is whether there are still opportunities for personal contact among providers. A personal handover was only described in some responses (8%). This agrees with another German study [25], in which the midwives rarely reported joint appointments and mainly experienced indirect transitions of care. However, face-to-face contact has been repeatedly described as promoting collaboration [3, 5], especially concerning care for families with high needs [4, 18]. It can also be an opportunity for less experienced professionals [25], is found enriching by service users [30] and could help to overcome communication challenges that have been repeatedly described [3, 4, 19]. A Swedish study by Barimani and Hylander suggests a possible explanation for these difficulties. Active collaboration initially means extra work for those early in the perinatal care chain [29]. In our case, family midwives or nurses are more dependent on independent midwives to provide care because they mostly provide care first. Interestingly, the same authors showed in a further study [30] that continuous joint work could eliminate this negative perception of collaboration. In the literature, it is also recommended that professionals get to know mutual roles [5] and make clear agreements [25], which requires contact between the professionals. In our survey, the meetings organised by the Early Prevention Networks were attended by one out of five midwives in the previous 12 months and played a minor role in transitions of care. One possible reason could be that network meetings only occur once or twice a year. Besides, independent midwives are not always invited [25]. These structures could therefore be improved to integrate more midwives.

### Self-assessment of knowledge around Early prevention

In this survey, three out of ten midwives reported feeling rather bad or very badly informed about data protection and confidentiality around Early Prevention. However, only 2.1% of responses (Fig. 3) explicitly attributed the absence of a transition of care to uncertainty or a lack of information. Still, a need for more training could be assumed, even if it rarely completely hinders the transition of cases.

As Schlüter-Cruse [25] described, some midwives might also experience difficulties handing the care to others, possibly leading to a higher workload and emotional burden. Some answers to the question about reasons for the absence of transition of care referred to the structural challenges of Early Prevention; these problems could be even more common among the participants because only the midwives without transition of care were asked this question. This result may underline that Early Prevention Network Services are still being implemented. However, the expansion suffers from structural problems: a lack of skilled care providers and financial resources [31]. The staff shortage also applies to independent midwifery care [7]. Thus, both care situations are interwoven. Staff shortages have been repeatedly described in the literature as inhibiting collaboration [3, 18, 25]. It also became clear that some midwives expressed a distanced attitude towards Early Prevention. Several participants stated that they did not consider Early Prevention to be helpful, they felt too insecure, or they needed to be more informed to establish contact. This again shows the necessity of promoting collaborative competencies.

In both questions on knowledge, it is noticeable that about one-third of the responses were negative. Even among those who selected the neutral option, one could assume a need for information. Thus, more than half of the participants did not answer ‘good’ for both questions. Furthermore, the fact that every fifth midwife in this survey could not name which institution is responsible for Early Prevention indicates a lack of essential preconditions for collaboration. This problem is already known: not surprisingly, a lack of information about professional partners may inhibit collaboration [3, 21]. Nevertheless, many midwives seemed satisfied with the collaboration: Three out of seven chose a positive answer, but only about one out of seven selected a negative one.

### Possible solutions to improve collaboration

When universal care by preventive maternal and child healthcare services is not offered, often perinatal healthcare professionals, such as independent midwives, must assess the need for further care and act as gatekeepers. Different understandings of Early Prevention could lead to heterogeneous assessments of care needs, which has already been found for paediatricians [8]. A systematic review [3] suggests that collegial consultation could facilitate these problems. However, when independent midwives provide their services as self-employed professionals, as in Germany [25], they must create their own structures for case consultation. Moreover, when team meetings are not remunerated, this may lead to additional burdens [21]. As a possible solution, integrated or co-located services have been established in several countries and are usually welcomed by parents and professionals [30]. These perinatal health centres can help to overcome fragmented services and inequalities, as they could offer an easily accessible point of contact for the target group and facilitate interdisciplinary care. Another option could be the integration of Early Prevention into community-based public health services so that family midwives and nurses work in the health sector. Family midwives are practising midwives and aim to promote maternal, infant and family health. Additionally, joint training would be a networking opportunity, as has been recommended [8]. In Germany, however, many training programmes are specific for each group. Joint home visits, also described as beneficial [5, 18, 25], could be better integrated into everyday life. They have the advantage of facilitating the exchange of information and offering an opportunity to learn about each other. Another promising approach is to provide users with more information about the organisation of perinatal healthcare, as has been suggested [18, 21]. Families would then be empowered in decision-making processes.

### Contextualisation of sample characteristics

In our sample, around 17% had an additional qualification which is granted with a certificate as a family midwife, higher than the 7–8% that were estimated in 2012 by the German Midwives Association [9], which points towards an increased interest in the topic. Unfortunately, there are no federal data on the number of midwives with other qualifications, such as a Bachelor’s, Master’s, or mentoring. The size of the municipalities where the midwives worked was similar to those found in a national database, with about a third working in large cities [32].

### Strengths and limitations of this survey

A major advantage of this work is examining midwifery-specific issues from the perspective of midwifery science, which facilitated field access and questionnaire design. Even though the potential of midwives as collaborative partners has been recognised [8], their perspective is sometimes neglected. Another strength of this work is that it explores a relatively unknown topic with a Germany-wide approach. Several publications are available internationally, but cannot always be applied to the German care system. Hence, it is preferable to use instruments adapted to the local context. Furthermore, the development of the questionnaire was supported by an extensive database search and a critical evaluation of the included literature. Care was also taken to test the questionnaire with members of the target group who did not later participate in the main survey. The Germany-wide sampling strategy with midwives from 13 of the 16 federal states and from supraregional organisations has its advantages because regional differences in perinatal care are known. In addition, the design of an online survey was appropriate for reaching many midwives in a short time.

An important first limitation is the possible selection bias. Midwives involved in professional associations might have been more willing to collaborate with others, and those interested in Early Prevention may have been more inclined to participate. Likewise, the electronic mailing of the questionnaire could have led to bias because it reaches more people with access and inclination to digital communication. Secondly, information bias may also have played a role. The participants might have problems recalling single cases or could answer in the direction of social desirability, for example, when asked about their knowledge. Another limitation is the participation. Although exact response rates could not be determined, it is clear that only a fraction of the addressees took part. A final limitation is that no validated questionnaire was available. However, we tested the questionnaire during a pilot phase to increase face validity. We did not assess the psychometric properties of the questionnaire including construct validity, reliability, and responsiveness, which could be relevant for future research.

### Perspectives for future midwifery research

Our findings suggest a possible subjective assessment of care needs and heterogeneous perceptions of Early Prevention Network Services among independent midwives. Further research could investigate their criteria for transitions of care. In addition, measures to promote collaboration should be developed and evaluated. This is especially true if independent midwifery care and Early Prevention remain separate systems.

In this sense, it would be interesting to evaluate model projects that provide integrated services for midwifery care, Early Prevention and other services for pregnant and childbearing women/people. It would be essential to include their perspectives, for instance, through focus groups. Psychosocial support by midwives is already an indispensable aspect of their work, but it deserves more recognition. Further investigation could strengthen this part of the midwives’ autonomous professional profile.

## Conclusion

The results support the need to improve collaboration with Early Prevention, at least for a part of the participating midwives. Further research can investigate targeted strategies to meet this. It is clear that establishing new forms of care, including successful collaboration, takes time. It is advantageous for this process that both professional groups pursue the same goal of quality care for mothers, babies and their families.

## Supplementary Information

Below is the link to the electronic supplementary material.

Supplementary Material 1

## Data Availability

The data protection requirements do not allow us to make the data publicly available. Please contact the corresponding author for reasonable requests and further questions about the data.
