# Supplementary material for: Transition from independent midwifery care to Early Prevention Network Services in Germany – a cross-sectional survey
Source: BMC Health Serv Res. 2026 Mar 24;26:493. doi: 10.1186/s12913-026-14414-4 (PMC13064050; doi:10.1186/s12913-026-14414-4)
Supplement: Supplementary file 1 — Supplementary Material 1 [file 12913_2026_14414_MOESM1_ESM.docx]

**S1. Questionnaire**

| 1. How many families have you supported as an independent midwife before, during and/or after childbirth in the last 2 months? |
| --- |
| Please enter the number: |
| 1. How many families have you supported as an independent midwife before, during and/or after childbirth in the last 12 months? |
| Please enter the number: |
| 1. Have you contacted the Early Prevention Network services in the last 12 months to find a family midwife or a family nurse, or have you successfully motivated the family to do so? |
| Yes  No |
| If yes: Questions 3.1-3.3, skip question 3.4.  If no: continue with question 3.4 |
| - 1. How many families have you contacted through the Early Prevention Network services in the last 2 months to arrange a family midwife or family nurse, or successfully motivated the family to do so? |
| Please enter the number: |
| - 1. How many families have you contacted through the Early Prevention Network services in the last 12 months to arrange a family midwife or family nurse, or successfully motivated the family to do so? |
| Please enter the number: |
| - 1. **How did these referrals mainly take place in the last 12 months? (Multiple answers possible)** |
| 1. The families contacted them themselves on my recommendation 2. Personal meeting or joint home visit with the family midwife or family nurse 3. Personal meeting or joint home visit with another Early Prevention staff member 4. By telephone or email to the family midwife or family nurse 5. By telephone or email to another Early Prevention staff member 6. Meeting with the Early Prevention Network 7. Referral via a specialist or institution from the health or social services sector that is not part of Early Prevention (e.g. clinic or paediatric practice) 8. Other (please specify): |
| - 1. **What are the reasons why you have not referred any families to a family midwife or family health and paediatric nurse in the last 12 months? (Multiple answers possible)** |
| 1. There is no Early Prevention network in the respective municipality 2. No Early Prevention capacities 3. The families did not need additional support 4. The families did not want additional support 5. I work with other partners in the health and social services sector who are not family midwives or family nurses 6. I do not believe that support from family midwives or family nurses is helpful for my clients 7. Other reasons (please specify): |
| 1. How large is the municipality in which you mainly work as an independent midwife? |
| 1. Large city (100,000 inhabitants and more) 2. Medium-sized city (between 20,000 and 99,999 inhabitants) 3. Small town (between 5,000 and 19,999 inhabitants) 4. Rural community (no town with at least 5,000 inhabitants) |
| 1. **Is there an early prevention network (a central institution that coordinates early prevention services for pregnant women and families) in the municipality where you most frequently work as an independent midwife?** |
| Yes  No |
| If yes: Questions 5.1-5.6  If no: continue with question 6 |
| - 1. **How long has this network been in existence?** |
| 1. 11-15 years 2. 6-10 years 3. 5 years or less 4. I don't know |
| - 1. Which institution is responsible for Early Prevention in the community where you most frequently work as a freelancer? |
| 1. Youth welfare office 2. Health authority 3. Independent or church-run organisation (such as Caritas or the Child Protection Agency) 4. I don't know |
| - 1. **Have you participated in any Early Prevention network meetings in the last 12 months?** |
| Yes  No |
| - 1. **How well informed do you feel about the organisation of Early Prevention in the local communities where you work?** |
| Very bad  Rather bad  Neither good nor bad  Rather good  Very good |
| - 1. **How satisfied are you with the cooperation with Early Prevention Network Services, including family midwives or family nurses in the municipality where you most frequently work as an independent midwife?** |
| Very dissatisfied  Dissatisfied  Neither satisfied nor dissatisfied  Satisfied  Very satisfied |
| - 1. **How well informed do you feel about data protection and confidentiality in relation to working with Early Prevention Services?** |
| Very bad  Rather bad  Neither good nor bad  Rather good  Very good |
| 1. **How old are you?** |
| Please enter the number: |
| 1. How much professional experience do you have as a midwife (in completed years)? |
| Please enter the number: |
| 1. **What qualifications do you have? (Multiple answers possible)** |
| 1. Mentor 2. Lead midwife 3. Family midwife 4. Bachelor's degree 5. Master's degree or higher academic qualification 6. fOther (please specify): |

**S2. Access routes for our participants (*n* = 292)**

| **Regions, organisations** | **Frequencies**  **n (%)** |
| --- | --- |
| *Regional midwifery organisations (*n*=261)* | |
| Bavaria | 15 (5 %) |
| Brandenburg | 3 (1 %) |
| Berlin | 2 (<1 %) |
| Bremen | 2 (<1 %) |
| Baden-Württemberg | 80 (27 %) |
| Hesse | 13 (4 %) |
| Lower Saxony | 17 (6 %) |
| North Rhine-Westphalia | 85 (29 %) |
| Rhineland-Palatinate | 5 (2 %) |
| Saarland | 2 (<1 %) |
| Saxony | 32 (11 %) |
| Schleswig-Holstein | 3 (1 %) |
| Thuringia | 2 (<1 %) |
| *Supraregional organisations (*n*=31)* | |
| Society for Quality in Out-of-Hospital Birth Care | 8 (3 %) |
| Facebook group for midwives | 11 (4 %) |
| Forum ‘www.hebammen.de’ | 8 (3 %) |
| Snowball principle | 4 (1 %) |

**S3. Size of municipality and transitions of care in past 12 months (*n* = 292)**

| **Size of municipality** | **Transitions of care in past 12 months**  **n (%)** | |
| --- | --- | --- |
|  | *Yes* | *No* |
| Large City (≥100,000 inhabitants) | 47 (35 %) | 53 (36%) |
| Medium-sized city (20,000–99,999 inhabitants) | 44 (33 %) | 40 (27%) |
| Town (5000–19,999 inhabitants) | 23 (17%) | 28 (19%) |
| Rural community | 20 (15 %) | 25 (17%) |
| Valid total (*n* = 280) | 134 | 146 |
| *12 missing observations* |  |  |

**S4. Care characteristics in past 2 months (N = 292)**

| **Characteristic** | ***n*** | **Missing (%)** | **Mean (SD)** | **Median (IQR)** | **Min–Max** |
| --- | --- | --- | --- | --- | --- |
| Families cared for in past 2 months | 286 | 6 (2.1) | 11.6 (8.8) | 10.0 (5.0–15.0) | 0–60 |
| Transitions of care in past 2 months | 291 | 1 (0.3) | 0.34 (0.7) | 0.0 (0.0–1.0) | 0–3 |

IQR: interquartile range; Min: minimum; Max: maximum; SD: standard deviation.
